# Exome Sequencing Resolving a Complex Pediatric Neurodevelopmental Disorder After Inconclusive Prenatal Testing: A Case Report

**DOI:** 10.3390/children13020202

**Published:** 2026-01-31

**Authors:** Margarita Panova, Hristo Ivanov, Iglika Sotkova-Ivanova

**Affiliations:** 1 Department of Pediatrics “Prof. Dr. Ivan Andreev”, Medical University Plovdiv, 4000 Plovdiv, Bulgaria; margarita.panova@mu-plovdiv.bg; 2 Department of Medical Genetics, Medical University Plovdiv, 4000 Plovdiv, Bulgaria; iglika.sotkova@mu-plovdiv.bg

**Keywords:** whole-exome sequencing, prenatal diagnosis, neurodevelopmental disorder, ventriculomegaly, *DDX3X*

## Abstract

**Highlights:**

**What are the main findings?**

- Prenatal chromosomal testing (karyotype, MLPA, and chromosome sequencing) may fail to detect monogenic causes of fetal central nervous system anomalies, even in the presence of ventriculomegaly.
- Trio-based whole-exome sequencing enabled the definitive postnatal diagnosis of a *DDX3X*-related neurodevelopmental disorder caused by a de novo pathogenic missense variant.

**What are the implications of the main findings?**

- Earlier selective implementation of prenatal exome sequencing in fetuses with complex or non-isolated CNS anomalies could significantly improve diagnostic yield and prognostic counseling.
- Integration of clinical genetics into multidisciplinary prenatal care pathways is essential to optimize decision-making, perinatal planning, and family counseling.

**Abstract:**

Background: Prenatal detection of fetal structural anomalies often prompts chromosomal analysis; however, chromosomal microarray analysis (CMA) has limited diagnostic yield for monogenic disorders. Whole-exome sequencing (WES) has emerged as a powerful tool for identifying single-gene etiologies, particularly in cases with complex neurodevelopmental phenotypes. Case Presentation: We report a female infant presenting with prenatally detected ventriculomegaly and inconclusive chromosomal testing. Prenatal investigations, including karyotyping and genome-wide chromosomal sequencing, identified several copy number variants classified as variants of uncertain significance but failed to establish a definitive diagnosis. Postnatally, the patient developed progressive neurological abnormalities, including microcephaly, facial dysmorphism, dystonic movements, and severe global developmental delay. Trio-based whole-exome sequencing identified a heterozygous de novo pathogenic missense variant in the *DDX3X* gene (c.976C>T; p.Arg326Cys), establishing the diagnosis of *DDX3X*-related neurodevelopmental disorder. Conclusions: This case highlights the diagnostic limitations of standard prenatal chromosomal testing in detecting monogenic neurodevelopmental disorders and underscores the critical role of timely genetic counseling and exome sequencing. Earlier selective implementation of WES during pregnancy could have enabled an earlier diagnosis, improved prognostic counseling, and optimized clinical decision-making.

## 1. Background and Clinical Significance:

Congenital anomalies are a leading cause of pediatric morbidity and mortality, with a substantial proportion attributable to pathogenic single-gene variants that remain undetectable by conventional cytogenetic methods. In pregnancies complicated by fetal structural anomalies, chromosomal microarray analysis (CMA) or chromosomal sequencing, performed by medium-coverage whole genome sequencing is widely recommended as a first-tier diagnostic test due to its ability to detect genome-wide copy number variants (CNVs) at high resolution [1,2,3,4,5]. However, CMA is inherently limited in its capacity to identify balanced rearrangements and single-nucleotide variants (SNVs) underlying monogenic disorders, and its diagnostic yield in fetuses with structural anomalies typically ranges from 4% to 20%, depending on the indication and analytical platform used [2,6].

Recent advances in next-generation sequencing have established whole-exome sequencing (WES) as a powerful approach for detecting sequence-level variants responsible for monogenic disease. The American College of Medical Genetics and Genomics and the American Academy of Pediatrics now recommend exome or genome sequencing as a first-tier diagnostic test for children with global developmental delay, intellectual disability, or congenital anomalies, citing improved diagnostic yield and cost-effectiveness when implemented early in the diagnostic pathway [1,7]. In the prenatal setting, WES provides an incremental diagnostic yield of approximately 10–40% in fetuses with structural anomalies and previously negative standard genetic testing, with the highest yields reported in central nervous system anomalies and multisystem involvement [6,8,9,10,11].

International professional societies, including the International Society for Prenatal Diagnosis and the Canadian College of Medical Geneticists, support the use of genome-wide sequencing in pregnancies complicated by multiple or severe fetal anomalies, emphasizing the critical role of detailed phenotyping and multidisciplinary genetic counseling [3,12].

Within this clinical and diagnostic framework, *DDX3X*-related neurodevelopmental disorder exemplifies the limitations of conventional prenatal testing strategies. The *DDX3X* gene is located on chromosome Xp11.4 and encodes a 662–amino acid DEAD-box RNA helicase that plays a central role in RNA metabolism, including transcriptional and translational regulation, pre-mRNA splicing, RNA stability, cell cycle control, and cellular stress responses [13]. The protein contains two highly conserved functional domains—the helicase ATP-binding domain and the helicase C-terminal domain—both essential for enzymatic activity and enriched for pathogenic missense variants. *DDX3X* is also a component of RNA–protein granules, including neuronal transport granules and cytoplasmic stress granules, underscoring its importance for neuronal homeostasis and brain development [13].

*DDX3X* is ubiquitously expressed across human tissues but plays a particularly critical role during embryogenesis and neurodevelopment. During fetal cortical development, *DDX3X* regulates neural progenitor proliferation, neuronal differentiation, and cortical lamination. Importantly, *DDX3X* exhibits sex-specific expression patterns [14]. The gene partially escapes X-chromosome inactivation in a context-dependent manner, resulting in higher overall *DDX3X* expression in females. Males possess a Y-linked paralog, *DDX3Y*, which shares high sequence similarity and partial functional redundancy with *DDX3X* but demonstrates more restricted and variable expression, with particularly limited expression in large regions of the central nervous system, contributing to sex-specific vulnerability to *DDX3X* loss [15].

Pathogenic variants in *DDX3X* cause *DDX3X*-related neurodevelopmental disorder (*DDX3X*-NDD; OMIM #300958). This condition represents one of the more frequent monogenic causes of neurodevelopmental impairment in females. To date, more than 200 affected individuals have been reported, with the largest published clinical cohort including over 100 patients [16]. While the postnatal phenotype of *DDX3X*-NDD is well characterized, prenatal imaging findings are less systematically documented; nevertheless, ventriculomegaly, corpus callosum abnormalities, and other central nervous system structural anomalies have been reported as presenting prenatal features [14].

The clinical phenotype is broad, typically including global developmental delay or intellectual disability, speech and language impairment, hypotonia, motor delay, movement disorders, and structural brain abnormalities such as ventriculomegaly, corpus callosum hypoplasia or dysgenesis, reduced white matter volume, and polymicrogyria [16]. Additional features may include microcephaly, epilepsy, autism spectrum disorder, attention-deficit/hyperactivity disorder, and multisystem involvement [16]. Genotype–phenotype correlations show that approximately half of pathogenic variants are missense mutations, often clustering in the helicase domains and associated with more severe neurological phenotypes, especially polymicrogyria [17]. Truncating variants support haploinsufficiency, while severe missense mutations may exert dominant-negative effects by disrupting RNA–protein granule formation and translation in neural progenitors [17]. *DDX3X*-NDD exhibits a striking female predominance, with most affected females harboring de novo pathogenic variants and low recurrence risk. Nevertheless, de novo pathogenic variants in males have also been reported [16].

In fetuses with ventriculomegaly and negative chromosomal testing, prenatal whole-exome sequencing has been shown to identify pathogenic single-gene variants in up to 48.5% of cases [18]. More broadly, meta-analyses of fetal central nervous system anomalies report pooled incremental diagnostic yields of approximately 38% for CNS anomalies overall and up to 46% for non-isolated CNS anomalies, representing the highest diagnostic yield among all fetal structural anomaly categories [9]. These data highlight the substantial diagnostic gap that remains when relying solely on conventional chromosomal testing strategies.

The diagnostic limitations of conventional prenatal testing are exemplified by *DDX3X*-related neurodevelopmental disorder, in which pathogenic de novo single-nucleotide variants are not detectable by karyotype or chromosomal microarray analysis but can be readily identified through trio-based whole-exome sequencing.

Early implementation of exome sequencing in pregnancies with complex neurodevelopmental phenotypes enables timely molecular diagnosis and informed prognostic counseling. When delivered within a multidisciplinary prenatal care team involving maternal–fetal medicine specialists, pediatric neurologists, radiologists, and clinical geneticists, this approach supports accurate interpretation of genomic findings and appropriate family counseling. This underscores the need for integrated prenatal care models that combine advanced genomic technologies with expert phenotyping and coordinated multidisciplinary evaluation. This case report does not aim to expand the phenotypic spectrum of *DDX3X*-related neurodevelopmental disorder, but rather to illustrate a diagnostic gap in prenatal genetic evaluation and the clinical consequences of delayed molecular diagnosis in the setting of fetal CNS anomalies.

## 2. Case Presentation

The patient is a female infant born from a third pregnancy to a 36-year-old mother (one healthy child and one previous spontaneous miscarriage). The pregnancy was initially uncomplicated. At the second-trimester detailed prenatal ultrasound anomaly scan (22 + 4 weeks of gestation), mild bilateral ventriculomegaly with visualization of the third ventricle was detected. No additional structural anomalies or indirect sonographic markers suggestive of chromosomal aneuploidy were identified. Maternal serological testing for Toxoplasma gondii, Epstein–Barr virus, and cytomegalovirus was negative.

**Figure 1:**
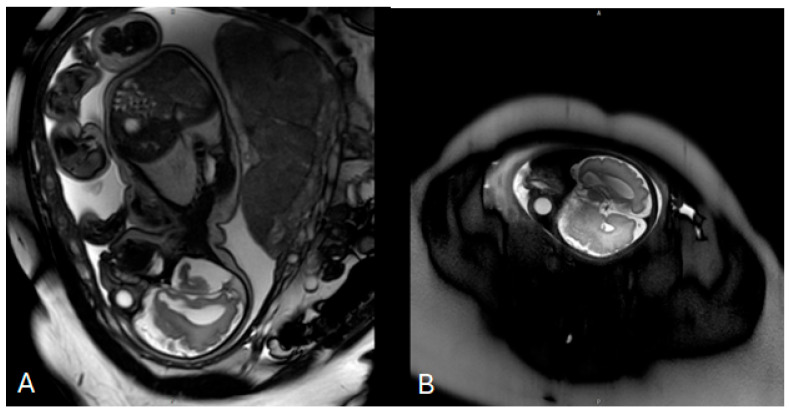
Prenatal fetal magnetic resonance imaging (MRI) performed at 33 weeks of gestation. (**A**) Axial T2-weighted image of the fetal brain demonstrating mild bilateral ventriculomegaly with visualization of the lateral ventricles. (**B**) Sagittal T2-weighted image confirming ventricular enlargement without additional major structural brain malformations at the time of examination.

Invasive prenatal genetic testing was performed via amniocentesis. Conventional cytogenetic analysis revealed a normal female karyotype (46,XX). Multiplex ligation-dependent probe amplification (MLPA) for microdeletion and microduplication screening yielded negative results. This was followed by genome-wide chromosome sequencing (ChromoSeq, BGI VISTA™), which identified four copy number variants, all classified as variants of uncertain significance. No pathogenic chromosomal aneuploidies or clinically relevant microdeletions or microduplications were detected. Fetal magnetic resonance imaging (MRI) performed at 33 weeks of gestation demonstrated mild ventriculomegaly without evidence of neural tube defects (Figure 1). No further genomic testing was pursued during pregnancy, and prenatal consultation with a clinical geneticist was not documented.

The infant was delivered at 39 weeks of gestation by vaginal delivery following a prolonged labor complicated by a nuchal cord. Apgar scores were 9 and 10 at 1 and 5 min, respectively. Birth parameters were within the normal range for gestational age, with a birth weight of 3180 g, length of 52 cm, and head circumference of 33 cm. The neonatal course was notable for delayed postnatal adaptation and feeding difficulties, as the infant did not latch effectively in the immediate postnatal period and required oxygen support for approximately 48 h. The patient was discharged on day four of life.

**Figure 2:**
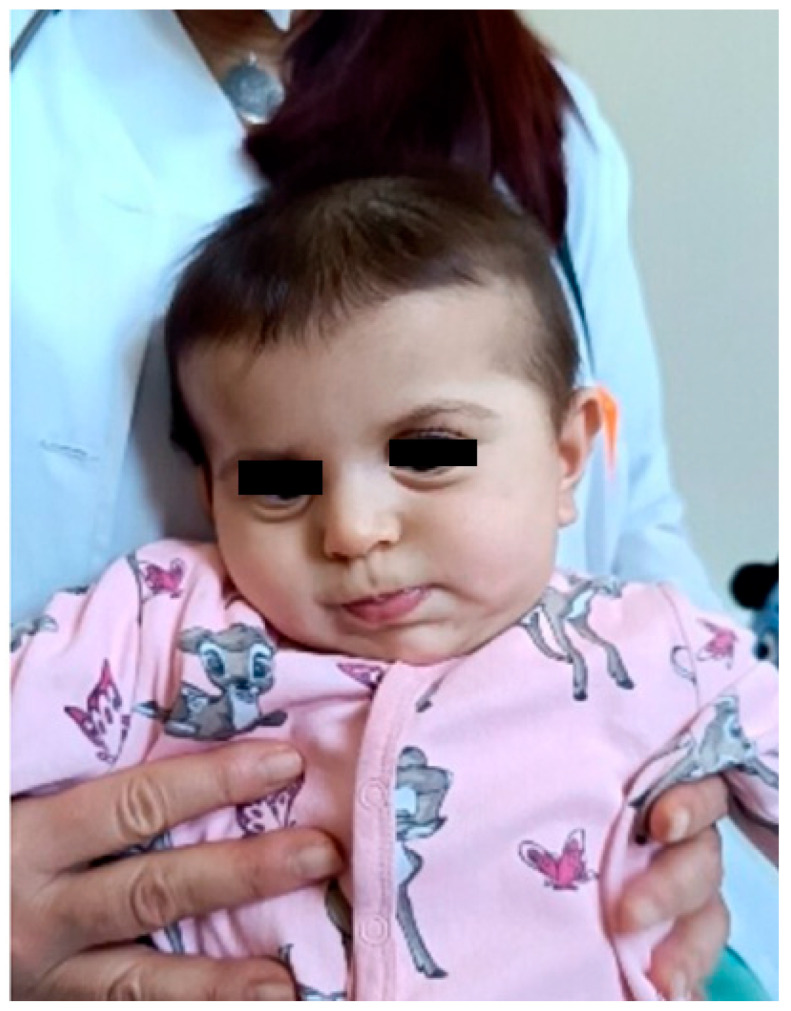
Clinical photograph of the patient in early infancy demonstrating facial dysmorphism, including relatively prominent eyes, full cheeks, an apparently shortened philtrum, a small mouth with full lips, and mildly low-set ears with subtle posterior rotation and simplified auricular morphology. The eyes are masked to protect patient privacy. These facial features are consistent with previously reported mild craniofacial dysmorphism in individuals with *DDX3X*-related neurodevelopmental disorder, including low-set or posteriorly rotated ears and subtle midline facial anomalies.

**Figure 3:**
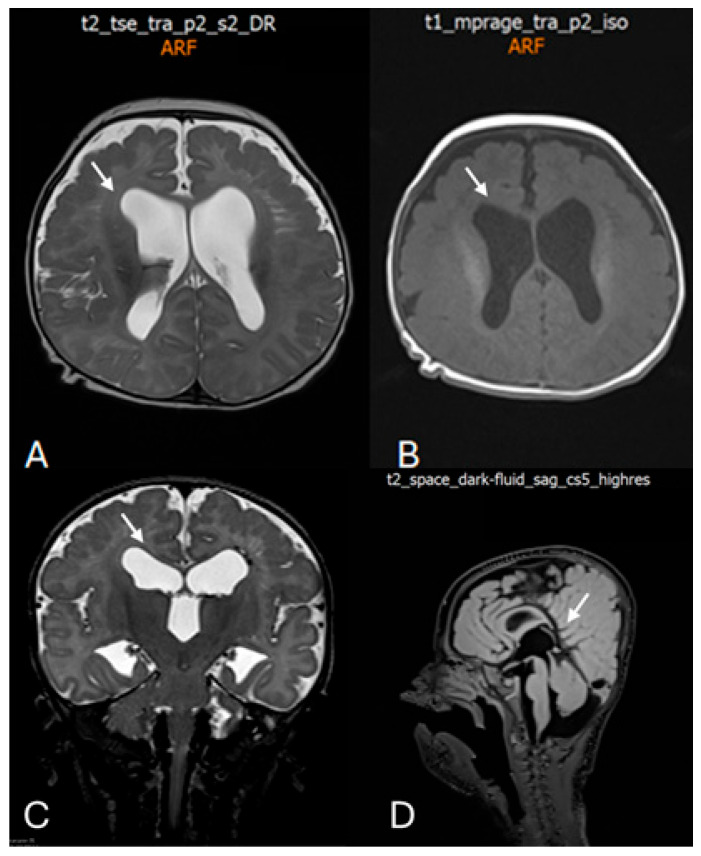
Postnatal brain magnetic resonance imaging (MRI) performed at 4 months of age. (**A**) Axial T2-weighted image demonstrating marked bilateral ventriculomegaly with symmetric dilatation of the lateral ventricles and reduced surrounding white matter volume. White arrow indicates enlargement of the lateral ventricles. (**B**) Axial T1-weighted image confirming ventriculomegaly and altered ventricular configuration (white arrow). (**C**) Coronal T2-weighted image showing symmetric enlargement of the lateral ventricles and dilatation of the third ventricle (white arrow). (**D**) Midline sagittal T2-weighted image illustrating the ventricular axis and involvement of the third ventricle (white arrow), providing additional characterization of the ventricular system.

At the age of 2 months and 20 days, the infant was evaluated for irritability and episodic opisthotonic posturing. Transfontanelle ultrasonography revealed ventriculomegaly with enlargement of the lateral and third ventricles. At 4 months of age, neurological examination demonstrated plagiocephaly, facial dysmorphism (Figure 2), and signs of a pyramidal syndrome affecting all four limbs. Brain MRI confirmed ventriculomegaly and additionally revealed chronic changes in the left periventricular region consistent with prior germinal matrix hemorrhage, as well as diffuse polymicrogyria predominantly involving the frontal lobes (Figure 3).

Over subsequent follow-up, the phenotype evolved toward severe neurodevelopmental impairment, characterized by progressive microcephaly, emerging dysmorphic facial features (relatively prominent eyes, full cheeks, an apparently shortened philtrum, a small mouth with full lips, and mildly low-set ears with subtle posterior rotation and simplified auricular morphology), and increasing motor abnormalities. Neurological examination demonstrated poor head control, axial hypotonia with evolving increased tone in the limbs, hyperreflexia with expanded reflexogenic zones, distal spasticity, and frequent hyperkinetic/dystonic movements, resulting in a cerebral palsy–like clinical presentation. Developmentally, the patient showed profound delay across all domains, with markedly delayed motor milestones and limited visual engagement.

**Figure 4:**
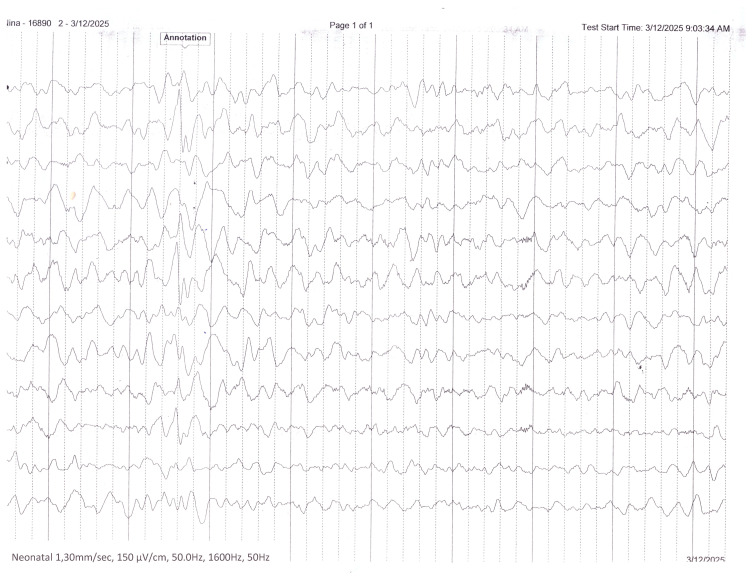
Interictal EEG recording performed during infancy demonstrates a disorganized background activity with multifocal epileptiform discharges.

At 7 months of age, the patient was admitted to a pediatric neurology unit for comprehensive evaluation. Clinical assessment confirmed progressive microcephaly, failure to thrive, and quadriparetic cerebral palsy with frequent hyperkinetic movements and dystonic episodes. Severe global developmental delay was evident, including absence of auditory fixation and profound impairment of both motor and cognitive milestones. Electroencephalography (EEG) (Figure 4) revealed abnormal background activity with intermittent focal slowing and suspicious epileptiform discharges; however, no unequivocal electroclinical seizures were recorded. In the absence of clear epileptic semiology and given the non-conclusive EEG findings, antiseizure therapy was deferred, with recommendations for video documentation of paroxysmal events and repeat EEG if episodes recurred. Abdominal ultrasound, echocardiography, and visual evoked potentials were normal. Given the complex neurodevelopmental phenotype and inconclusive prenatal genetic investigations, trio-based whole-exome sequencing was performed postnatally. Analysis using the Blueprint Genetics Whole-Exome Family Plus approach identified a heterozygous de novo missense variant in the *DDX3X* gene: c.976C>T (p.Arg326Cys), thereby establishing the molecular diagnosis of *DDX3X*-related neurodevelopmental disorder (OMIM #300958).

## 3. Genetic Findings

Trio-based whole-exome sequencing (WES) was performed on the patient and both parents using the Blueprint Genetics Whole-Exome Family Plus approach. Sequencing and variant analysis focused on genes associated with neurodevelopmental disorders and congenital brain malformations, using a phenotype-driven approach. Variant interpretation was conducted according to the American College of Medical Genetics and Genomics (ACMG) and Association for Molecular Pathology (AMP) guidelines.

A heterozygous missense variant was identified in the *DDX3X* gene: c.976C>T, resulting in the amino acid substitution p.Arg326Cys. The variant was confirmed to have arisen de novo, as it was absent in both parents. It is not present in population reference databases, including gnomAD, supporting its rarity.

The affected residue is highly conserved and located within the helicase ATP-binding domain of the *DDX3X* protein, a region critical for RNA metabolism and neurodevelopmental processes. In silico prediction tools consistently supported a deleterious effect on protein function. Notably, the same variant has been previously reported in multiple unrelated female individuals with *DDX3X*-related neurodevelopmental disorder, and another missense substitution affecting the same codon has also been associated with a similar phenotype, indicating functional importance of this amino acid position.

Based on ACMG/AMP criteria, the variant was classified as pathogenic, supported by strong evidence of de novo occurrence (PS2), absence from controls (PM2), multiple independent reports in affected individuals (PS4), and consistent computational and functional domain-based evidence (PP3).

The molecular findings established the diagnosis of *DDX3X*-related neurodevelopmental disorder, explaining the patient’s complex neurological phenotype, including ventriculomegaly, cortical malformations, progressive microcephaly, severe global developmental delay, and movement disorder.

## 4. Discussion

The novelty of this case lies not in the identification of a novel variant or an unusual phenotype, but in demonstrating how the timing of genomic testing directly influenced prenatal counseling and clinical decision-making.

This case further highlights the diagnostic limitations of conventional prenatal genetic testing in pregnancies complicated by fetal central nervous system (CNS) anomalies and underscores the clinical value of timely genome-wide sequencing. Despite early sonographic detection of ventriculomegaly and the application of standard invasive investigations, including karyotyping, MLPA-based microdeletion screening, and genome-wide chromosome sequencing, a definitive molecular diagnosis was not achieved during pregnancy.

Accumulating evidence demonstrates that chromosomal testing approaches, while essential first-tier investigations, have limited diagnostic utility for monogenic neurodevelopmental disorders. In contrast, prenatal whole-exome sequencing (WES) provides a substantially higher diagnostic yield in fetuses with CNS anomalies following negative karyotype and chromosomal microarray analysis, with reported yields ranging from approximately 26.5% to 46% [6,9,13,19]. Notably, ventriculomegaly represents one of the prenatal phenotypes with the highest diagnostic yield, with recent studies identifying pathogenic single-gene variants in up to 48.5% of copy number–negative cases, particularly when ventriculomegaly is non-isolated or associated with complex brain malformations [6,9,13,19]. Diagnostic yields are consistently higher for CNS anomalies compared with other fetal structural abnormalities, with isolated CNS anomalies yielding approximately 22%, CNS-only multiple anomalies 33%, and non-isolated CNS anomalies up to 46% [9,13,19].

*DDX3X*-related neurodevelopmental disorder exemplifies the limitations of copy number-based approaches in this clinical context. Pathogenic variants in *DDX3X* are predominantly de novo single-nucleotide variants, most often missense or truncating, and therefore remain undetectable by karyotype or copy number-focused approaches. Prenatal and postnatal neuroimaging studies have demonstrated a characteristic spectrum of brain malformations associated with *DDX3X* variants, including ventriculomegaly, corpus callosum hypoplasia or dysgenesis, polymicrogyria, reduced white matter volume, and abnormalities of the brainstem and cerebellum [14,20,21]. Missense variants affecting the helicase domains, particularly recurrent dominant mutations, are strongly associated with polymicrogyria and severe neurological impairment, consistent with the severe phenotype observed in the present patient [14,20,21].

Beyond establishing a molecular diagnosis, prenatal genomic testing has broad implications for long-term clinical management and family-centered care. Prenatal implementation of WES enables earlier molecular diagnosis, facilitating accurate prognostic assessment, recurrence risk counseling, and informed pregnancy management decisions [22,23,24]. In cases of severe or complex CNS anomalies, some experts now advocate for exome sequencing as a first-tier test, given its ability to detect both pathogenic sequence variants and copy number variants within the time constraints of an ongoing pregnancy [22,23,24]. Early genomic diagnosis further enables anticipatory perinatal care planning, targeted postnatal surveillance, cascade testing of family members, and informed reproductive decision-making for both current and subsequent pregnancies. Previous studies have demonstrated that genomic diagnoses could be established years earlier if sequencing were implemented at the onset of clinical suspicion, thereby avoiding prolonged diagnostic odysseys and their associated psychosocial burden [12,22,23,25,26]. Moreover, a significant proportion of prenatally diagnosed cases harbor inherited pathogenic variants, emphasizing the importance of timely diagnosis for accurate recurrence risk assessment in future pregnancies [23,24].

This case further underscores the critical importance of multidisciplinary prenatal care and early involvement of a clinical geneticist in the diagnostic pathway. In the present patient, the absence of documented prenatal genetic counseling and delayed implementation of genome-wide sequencing contributed to a postponed molecular diagnosis. A structured multidisciplinary approach, guided by detailed fetal phenotyping and comprehensive pre- and post-test counseling, is essential to ensure accurate interpretation of genomic findings and to maximize both clinical and personal utility for affected families [12,26].

From a clinical practice perspective, amniocentesis with chromosomal microarray analysis or chromosomal sequencing should remain the initial diagnostic step when ventriculomegaly is detected. However, clinicians must explicitly counsel families regarding the limitations of CMA in detecting monogenic disorders. For fetuses with CNS anomalies and non-diagnostic standard testing, trio-based WES should be strongly considered, particularly in cases with multiple CNS findings, complex phenotypes, or recurrent adverse pregnancy outcomes. Recent systematic reviews and professional society recommendations support the integration of WES into prenatal diagnostic algorithms for fetal CNS anomalies to enable accurate prognostic counseling and informed decision-making [9,12,24,25,26,27].

In the present case, earlier implementation of prenatal WES in the setting of fetal ventriculomegaly could have enabled prenatal diagnosis of *DDX3X*-related neurodevelopmental disorder. This would have facilitated comprehensive counseling regarding prognosis, recurrence risk, and pregnancy management options. Collectively, these findings reinforce the need for early involvement of clinical genetics specialists and systematic integration of advanced genomic technologies into prenatal diagnostic pathways for fetal CNS anomalies.

Earlier prenatal molecular diagnosis in this case would not have altered postnatal treatment options, but it would have enabled comprehensive prognostic counseling and informed reproductive decision-making, including pregnancy management options in accordance with parental values and local legal regulations.

## 5. Conclusions

This case demonstrates that conventional prenatal genetic testing, while essential, may be insufficient for establishing a molecular diagnosis in fetuses with complex central nervous system anomalies. *DDX3X*-related neurodevelopmental disorder exemplifies a class of monogenic conditions in which pathogenic single-nucleotide variants are not detectable by karyotype, MLPA-based screening, or copy number-focused approaches, yet can be readily identified by whole-exome sequencing.

Accumulating evidence indicates that in selected high-risk scenarios—particularly fetuses with ventriculomegaly, cortical malformations, or non-isolated CNS anomalies—prenatal exome sequencing provides a superior diagnostic yield compared with stepwise chromosomal testing strategies. In such contexts, early implementation of trio-based WES may represent the most efficient diagnostic approach, enabling timely molecular diagnosis, accurate prognostic counseling, and informed pregnancy and perinatal management.

The present case supports earlier integration of exome sequencing into the prenatal evaluation of fetal CNS anomalies, provided that testing is performed within a multidisciplinary clinical framework and accompanied by expert genetic counseling. Early involvement of a clinical geneticist and coordinated multidisciplinary care are critical to maximizing both the clinical and personal utility of genomic findings. Early implementation of exome sequencing in pregnancies complicated by complex neurodevelopmental phenotypes enables timely molecular diagnosis, informed prognostic counseling, and optimized clinical decision-making when delivered within a multidisciplinary prenatal care framework involving maternal–fetal medicine specialists, pediatric neurologists, radiologists, and clinical geneticists. This approach ensures accurate interpretation of genomic findings and appropriate counseling of affected families, underscoring the need for integrated prenatal care models that combine advanced genomic technologies with expert phenotyping and coordinated multidisciplinary evaluation.

## Data Availability

Data are available upon reasonable request from the corresponding author.

## Abbreviations

The following abbreviations are used in this manuscript:

ACMG	American College of Medical Genetics and Genomics
AMP	Association for Molecular Pathology
aCGH	Array Comparative Genomic Hybridization
BGI	Beijing Genomics Institute
CMA	Chromosomal Microarray Analysis
CNS	Central Nervous System
CNV	Copy Number Variant
EEG	Electroencephalography
ES	Exome Sequencing
gnomAD	Genome Aggregation Database
IRB	Institutional Review Board
MLPA	Multiplex Ligation-Dependent Probe Amplification
MRI	Magnetic Resonance Imaging
SNV	Single-Nucleotide Variant
VUS	Variant of Uncertain Significance
WES	Whole-Exome Sequencing
